# Identification of Iron Metabolism-Related Gene Signatures for Predicting the Prognosis of Patients With Sarcomas

**DOI:** 10.3389/fonc.2020.599816

**Published:** 2021-01-07

**Authors:** Jianyi Li, Chuan Hu, Yukun Du, Xiaojie Tang, Cheng Shao, Tongshuai Xu, Zheng Zhao, Huiqiang Hu, Yingyi Sheng, Jianwei Guo, Yongming Xi

**Affiliations:** ^1^ Department of Orthopaedic Surgery, The Affiliated Hospital of Qingdao University, Qingdao, China; ^2^ Department of Spinal Surgery, Yantai Affiliated Hospital of Binzhou Medical University, Yantai, China

**Keywords:** iron metabolism-related genes, prognostic signature, nomogram, sarcoma, The Cancer Genome Atlas (TCGA)

## Abstract

Iron is one of the essential trace elements in the human body. An increasing amount of evidence indicates that the imbalance of iron metabolism is related to the occurrence and development of cancer. Here, we obtained the gene expression and clinical data of sarcoma patients from TCGA and the GEO database. The prognostic value of iron metabolism-related genes (IMRGs) in patients with sarcoma and the relationship between these genes and the immune microenvironment were studied by comprehensive bioinformatics analyses. Two signatures based on IMRGs were generated for the overall survival (OS) and disease-free survival (DFS) of sarcoma patients. At 3, 5, and 7 years, the areas under the curve (AUCs) of the OS signature were 0.708, 0.713, and 0.688, respectively. The AUCs of the DFS signature at 3, 5, and 7 years were 0.717, 0.689, and 0.702, respectively. Kaplan–Meier survival analysis indicated that the prognosis of high-risk patients was worse than that of low-risk patients. In addition, immunological analysis showed that there were different patterns of immune cell infiltration among patients in different clusters. Finally, we constructed two nomograms that can be used to predict the OS and DFS of sarcoma patients. The C-index was 0.766 (95% CI: 0.697–0.835) and 0.763 (95% CI: 0.706–0.820) for the OS and DFS nomograms, respectively. Both the ROC curves and the calibration plots showed that the two nomograms have good predictive performance. In summary, we constructed two IMRG-based prognostic models that can effectively predict the OS and DFS of sarcoma patients.

## Introduction

Sarcomas are extremely rare malignancies of mesenchymal origin with high heterogeneity, and they account for approximately 1% of adult malignancies (1). It is estimated that the total incidence of sarcomas in EU countries is 5.6 per 100,000 (2). At present, more than 70 histological subtypes of sarcoma have been identified, and they can occur in different anatomical locations. Sarcomas can be divided into two categories: soft tissue sarcoma (STS), which accounts for 80% of sarcomas, and osteosarcoma (3). Due to the characteristics of aggressive growth and a high risk of metastasis, the prognosis of sarcoma patients is unsatisfactory (4). Consequently, it is vital to develop new biomarkers for accurately predicting the prognosis of sarcoma patients.

Iron is an essential element for cells to maintain normal function and homeostasis. The imbalance of iron metabolism is closely related to the occurrence, development and metastasis of tumors (5–7). Notably, iron metabolism has dual effects in tumor cells. On the one hand, the proliferation of tumor cells is more dependent on iron than that of normal cells, a phenomenon known as iron addiction (8). On the other hand, increased iron concentrations cause cell death through the accumulation of reactive oxygen species (ROS) and lipid peroxidation products, termed ferroptosis (9, 10). Ferroptosis is a new type of programmed cell death that is different from apoptosis, cell necrosis, and autophagy (11). As emerging anticancer pathways have been studied, a variety of ferroptosis inducers have been developed for the treatment of cancer (12, 13).

In the present study, we conducted extensive analysis based on transcript and clinical data obtained from The Cancer Genome Atlas (TCGA) and the Gene Expression Omnibus (GEO) database. We applied consensus clustering analysis, least absolute shrinkage and selection operator (LASSO) regression analysis, and Cox regression analysis to develop two prognostic iron metabolism-related gene (IMRG) signatures. To further explore the potential relationship between IMRGs and clinicopathological data, we developed two clinical IMRG nomograms to predict the prognosis of and to suggest therapeutic targets for sarcoma patients.

## Materials and Methods

### Data Sources

Seventy IMRGs were collected from the published literature. RNA-seq transcriptome and clinical data sets were obtained from TCGA (https://portal.gdc.cancer.gov/). Patients with unclear survival time, survival status, and clinicopathological characteristics were excluded. Additionally, the gene expression profile and clinical data of the two independent cohorts, GSE17674 and GSE30929, were obtained from the GEO database (https://www.ncbi.nlm.nih.gov/geo/) as the external validation cohorts. Among them, the GSE17674 cohort was used to verify the overall survival (OS) signature, while the GSE30929 cohort was used to validate the disease-free survival (DFS) signature. All data were collected on 18 April 2020.

### Comprehensive Analyses of Iron Metabolism-Related Gene-Based Clusters in Sarcoma Patients

Based on the expression pattern of IMRGs, 259 patients with sarcoma were classified as unsupervised by the “ConensusClusterPlus” software package, and unbiased and unsupervised outcomes were obtained. Second, using the “limma” software package, the accuracy of the clustering results was verified by principal component analysis (PCA). The survival software package was used to analyze the difference in DFS in different clusters of sarcoma. To further explore the difference in the tumor microenvironment (TME) among different clusters based on the above tumor classification, by performing ESTIMATE and CIBERSORT in R, the TME scores and the fraction of 21 types of immune cells were determined. The differences in prognosis, TME score, and immune cells were assessed using the Wilcoxon rank-sum test or the Kruskal–Wallis test.

### Construction and Validation of Iron Metabolism-Related Gene Signature

To identify the prognostic IMRGs, using the R software package “survival”, we first performed univariate Cox regression analysis and then used the machine learning algorithm LASSO regression analysis to further eliminate overfitting. Finally, the genes that can be used as independent prognostic factors of OS and DFS were screened by multivariate Cox regression analysis, and their regression coefficients (*β*) were calculated. The risk score of each sample was calculated, and the formula was as follows:

$$\mathrm{Risk score}\;=\;expr_{\text{gene}1}\;*\;\beta_{\text{gene}1}\;+\;\text{exp}{\text{r}}_{\text{gene}2}\;*\;\beta_{\text{gene}2}+\;\text{exp}{\text{r}}_{\text{gene}3}\;*\;\beta_{\text{gene}3}\ldots \ldots \text{exp}{\text{r}}_{\text{gene}n}\;*\;\beta_{\text{gene}\;\text{n}}$$

Subsequently, all patients were divided into high-risk and low-risk groups based on the median risk score. The Kaplan–Meier method was performed to compare the survival difference between two risk subgroups. The prediction accuracy of the multi-gene signature was assessed by receiver operating characteristic (ROC) analysis.

In addition, to ensure the stability of the two prognostic signatures, we calculated the risk score of patients in two validation cohorts. The Kaplan–Meier survival curve and survival ROC curve were developed to show the predictive ability of prognostic signatures in the validation cohorts.

### Establishment of a Clinical Iron Metabolism-Related Gene Nomogram for Sarcoma Patients

Nomograms are a visual clinical predictive model tool that is widely used to evaluate the prognosis of cancer patients. Therefore, we developed a nomogram based on the prognostic signature of IMRGs and clinicopathological data to predict the prognosis of patients with sarcoma. First, we performed univariate Cox regression analysis to evaluate the prognostic value of polygenic signatures and clinicopathological features. Multivariate Cox regression analysis was used to further determine the independent prognostic factors. Afterward, two nomograms were established by the “rms” package for predicting OS and DFS. Finally, the C-index, and calibration plot were constructed to estimate the accuracy and consistency of the prognostic models.

### Statistical Analysis

SPSS 21.0 (SPSS Inc., Chicago, IL, USA) and R software (version 3.6.1) were used for all statistical analyses. Univariate and multivariate Cox regression analyses, ROC curve analysis and K–M survival analysis were performed by R software and the corresponding R packages. The continuous data are expressed as the mean ± standard deviation (SD). The Wilcoxon test was used for comparisons between the two groups, and the Kruskal–Wallis test was used for comparisons of prognoses between groups. Except for the special instructions, all statistical tests were two-tailed, and a P <0.05 was considered to be statistically significant.

## Results

### Overview of Survival Data of Sarcoma Patients

According to the aforementioned criteria, 259 patients with primary sarcoma participated in this study, including 118 males and 141 females. The mean age was 60.71 ± 14.59. A total of 231 patients with DFS data were used to study DFS-related genes, including 108 males and 123 females, and the mean age was 60.09 ± 14.65. The demographic and clinicopathological data included in the sample are shown in **Supplementary File 1**.

### Iron Metabolism-Related Gene-Based Clusters Were Significantly Associated With Immune Function

**Figure 1:**
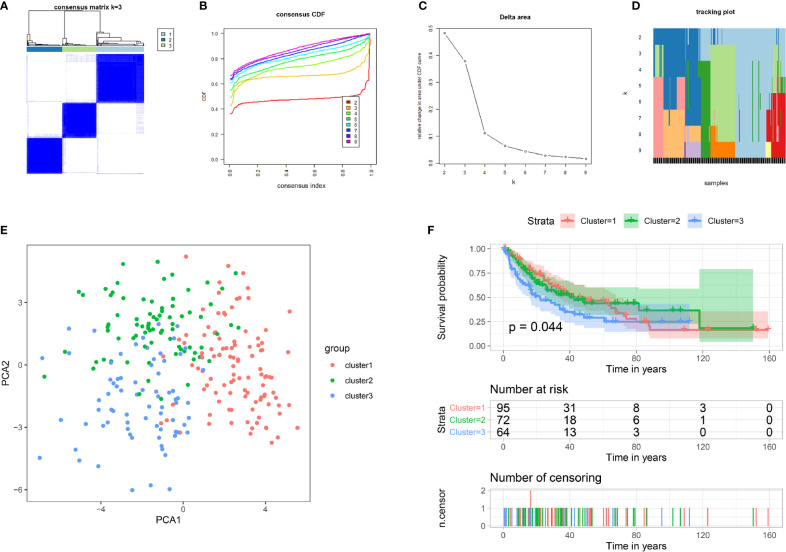
Tumor classification and verification based on IMRGs. **(A–D)** Unsupervised clustering of all samples based on the IMRGs. **(E)** PCA based on clustering results. **(F)** Kaplan**–**Meier survival analysis of DFS in different subgroups. IMRGs, iron metabolism-related genes; PCA, principal component analysis; DFS, disease-free survival.

To gain insight into the molecular heterogeneity of STS and explore whether IMRGs presented discernible patterns in sarcoma, we performed unsupervised consensus analysis of all samples. The result of k = 3 seemed to be more accurate, which could divide all samples into three groups with less correlation between groups (**Figures 1A–D**). Next, we performed PCA to further show the effect of distinction on the transcriptional profile between cluster 1, cluster 2, and cluster 3 (**Figure 1E**). To explore whether there was a correlation between the clustering result and clinical outcome, we compared the DFS among the three clusters of patients *via* the Kaplan–Meier analysis. The results showed that patients in the cluster 3 subgroup had shorter DFS (p = 0.044) than the other two clusters (**Figure 1F**).

**Figure 2:**
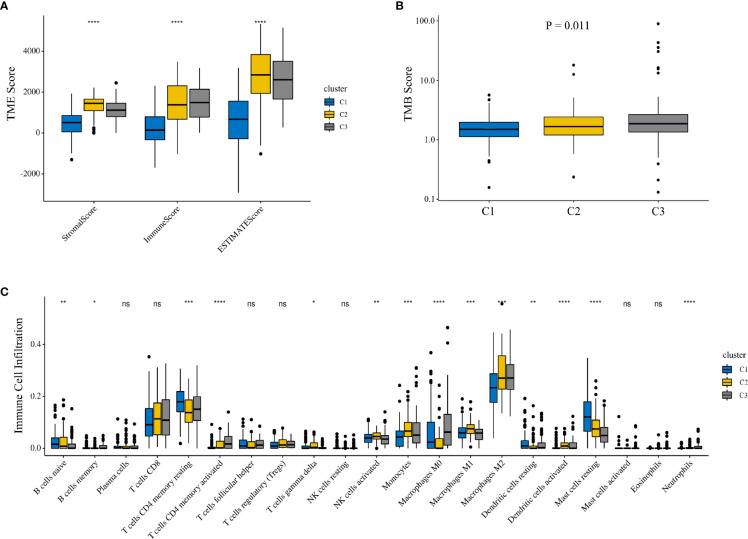
Comparison of the TME among different clusters. **(A)** The Kruskal**–**Wallis test was used to compare the TME-related scores between the three clusters. **(B)** Comparison of TMB among three clusters. **(C)** Comparison of 21 immune cells between the three clusters. TME, tumor microenvironment; TMB, tumor mutation burden.

To investigate whether there was a difference in the TME between different clusters, we employed the Kruskal–Wallis test to compare the scores related to the TME between the three clusters (**Figure 2A**). As the results showed, the three clusters showed significant differences in the scores of the three TMEs, including stromal (p < 0.001), immune (p < 0.001), and ESTIMATE (p < 0.001) microenvironments. In addition, we compared the differences in tumor mutation burden (TMB) between different clusters. The results showed a significant difference in TMB (p = 0.011), and cluster 3 had the highest TMB compared with the other clusters (**Figure 2B**). To assess the correlation between IMRGs and additional immune infiltration characteristics, we compared the levels of 21 types of immune cells among the three clusters (**Figure 2C**). The results revealed that the expression levels of naive B cells, memory B cells, resting memory CD4 T cells, activated memory CD4 T cells, delta gamma T cells, activated NK cells, monocytes, M0 macrophages, M1 macrophages, M2 macrophages, resting dendritic cells, activated dendritic cells and resting mast cells were significantly different among the three clusters.

### Construction and Validation of an Iron Metabolism-Related Gene Signature for Overall Survival

**Figure 3:**
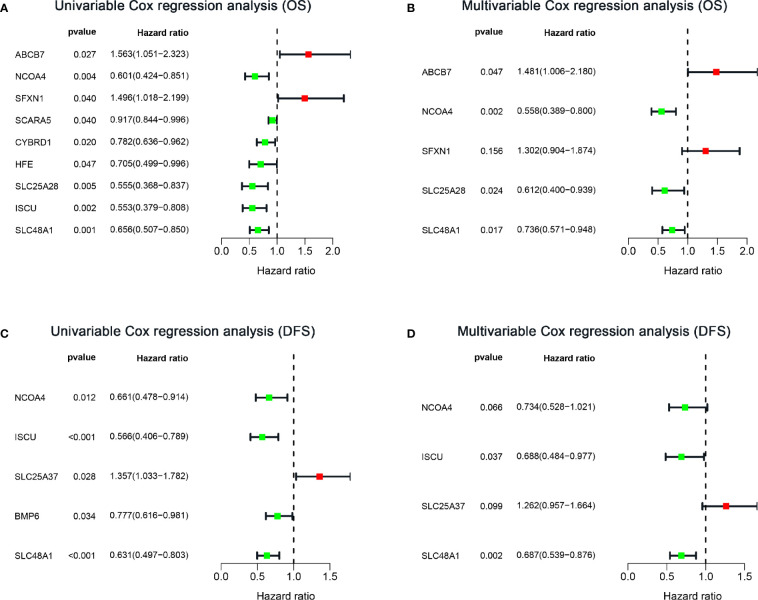
Forest plots of IMRG hazard ratios of prognosis-associated IMRGs in sarcoma patients. **(A, B)** Univariate and multivariate Cox analysis results of OS-related IMRGs. **(C, D)** Univariate and multivariate Cox analysis results of DFS-related IMRGs. OS, Overall survival; DFS, disease-free survival; IMRGs, iron metabolism-related genes.

To explore the prognostic role of IMRGs in sarcomas, we first performed univariate Cox regression analysis to identify genes associated with OS in the training set (**Figure 3A**). Nine IMRGs were selected. Then, we performed LASSO regression analysis and stepwise multivariate Cox regression analysis to establish an optimal multigene prognostic signature for OS, which was composed of ABCB7, NCOA4, SFXN1, SLC25A28, and SLC48A1 (**Figures S1A, B**, **Figure 3B** and **Supplementary File 2**). The risk coefficients generated by the multivariate Cox regression analysis were used to calculate the risk score of each patient in the training and validation sets. The formula for calculating the risk scores was as follows:

risk score = (0.393) ∗ ABCB + (0.584) ∗ NCOA4 + (0.264) ∗ SFXN1 + (−0.490) ∗ SLC25A28 + (-0.307) ∗ SLC48A1

**Figure 4:**
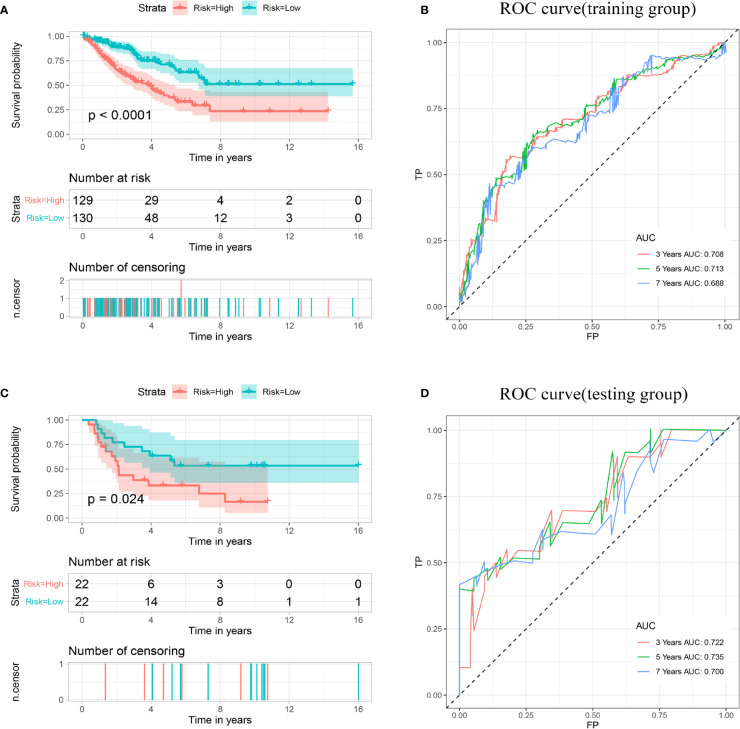
Establishment and validation of a prognostic model related to OS based on IMGRs. **(A)** The survival curve shows that the OS status of high-risk and low-risk patients in the training cohort is different. **(B)** Receiver operating characteristic curve of the prognostic signature in the training cohort. **(C)** The survival curve shows that the OS status of high-risk and low-risk patients in the validation cohort is different. **(D)** Receiver operating characteristic curves of the prognostic signature in the validation cohort. OS, Overall survival; IMRGs, iron metabolism-related genes.

Based on the median risk scores, the patients in the training and validation sets were divided into high and low groups. To determine whether the multigene signature can accurately predict the prognosis of patients with sarcomas, the Kaplan–Meier method was conducted (**Figures 4A, C**). The results demonstrated that patients in the high-risk group had lower OS than patients in the low-risk group (p < 0.001), consistent with the results obtained in the validation set (p < 0.001). The AUCs for 3-, 5- and 7-year OS shown by ROC analysis reached 0.708, 0.713, and 0.688 in the training cohort and 0.722, 0.735, and 0.700 in the validation cohort, respectively (**Figures 4B, D**). These results revealed that the prognostic signature for OS could effectively screen out high-risk sarcoma patients with relatively worse OS.

### Construction and Validation of an Iron Metabolism-Related Gene Signature for Disease-Free Survival

Considering the importance of DFS in the clinical outcome of patients, we also constructed a prognostic IMRG signature for DFS. After univariate Cox regression analysis, five IMRGs were found to be associated with DFS in sarcoma patients (**Figure 3C**). After LASSO regression analysis and stepwise multivariate Cox regression analysis (**Figures S1C, D**, **Figure 3D** and **Supplementary File 3**), we finally obtained four IMRGs and established a multigene prognostic signature. Based on the coefficients, the risk score of each patient was calculated, and the formula was as follows:

risk score = (−0.309) ∗ NCOA4 + (−0.374) ∗ ISCU + (0.233) ∗ SLC25A37 + (−0.375) ∗ SLC48A1

**Figure 5:**
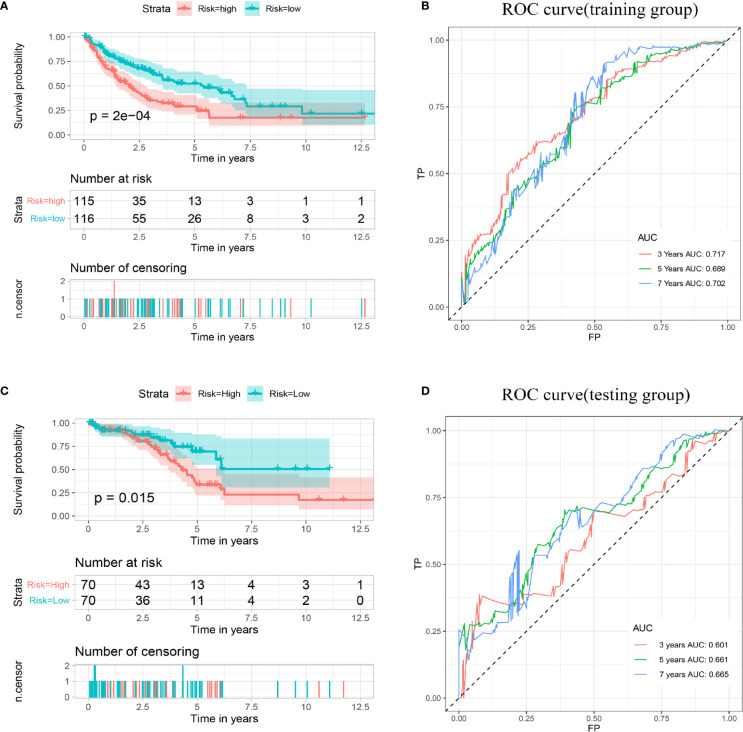
Establishment and validation of a prognostic model related to DFS based on IMGRs. **(A)** The survival curve shows that the DFS status of high-risk and low-risk patients in the training cohort is different. **(B)** Receiver operating characteristic curve of the prognostic signature in the training cohort. **(C)** The survival curve shows that the DFS status of high-risk and low-risk patients in the validation cohort was different. **(D)** Receiver operating characteristic curves of the prognostic signature in the validation cohort. DFS, disease-free survival; IMRGs, iron metabolism-related genes.

Then, according to the median risk score, all patients in the training cohort and validation cohort were divided into high- and low-risk groups. The Kaplan–Meier analysis showed that patients in the high-risk group had a relatively shorter DFS (p < 0.001). Consistent results were also found in the validation cohort (**Figures 5A, C** **)**. The AUCs for 3-, 5- and 7-year DFS were 0.717, 0.689, and 0.702 in the training cohort, respectively, and 0.601, 0.661, and 0.664 in the validation cohort, respectively (**Figures 5B, D**). These results indicated that the multigene prognostic signature for DFS can also accurately predict the clinical outcome of sarcoma patients.

### Development of Nomogram of Patients With Sarcoma Based on OS and DFS

**Figure 6:**
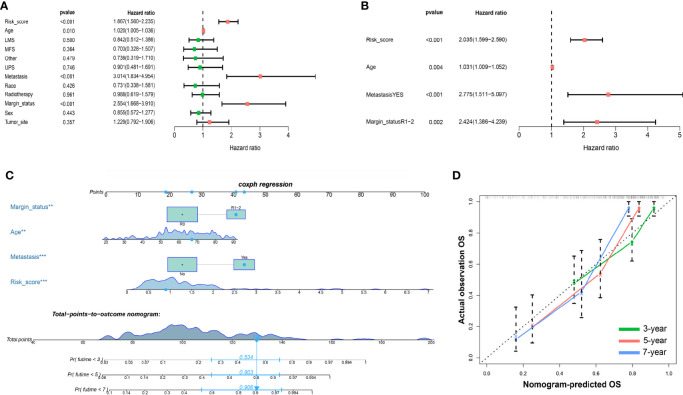
Nomograms based on the OS-related IMRGs for osteosarcoma patients. **(A)** Univariate Cox analysis of OS-related variables. **(B)** Multivariate Cox analysis of OS-related variables. **(C)** Establish a nomogram to predict the OS of patients. **(D)** The calibration curve shows that using a nomogram to predict OS is highly consistent with actual OS. OS, Overall survival; IMRGs, iron metabolism-related genes.

To confirm whether the iron metabolism-related signature for OS was an independent prognostic factor, univariate and multivariate Cox regression analyses were performed (**Figures 6A, B**). As the results showed, in the univariate Cox regression analysis, risk score, age, metastasis and margin status were significantly associated with the OS of sarcoma patients. Then, risk score, age, metastasis and margin status were identified as independent prognostic factors of sarcomas *via* multivariate Cox regression analysis. All independent factors were combined to establish a nomogram for predicting the 3-, 5- and 7-year OS (**Figure 6C**). As shown in **Figure 6**, the risk score contributes more to the total score than other variables. The 3-, 5-, and 7-year OS rates of patients declined as the total score increased. The C-index reached 0.766 (95% CI: 0.697–0.835). The calibration plots approached 45 degrees (**Figure 6D**). These results indicated that the nomogram had great performance.

**Figure 7:**
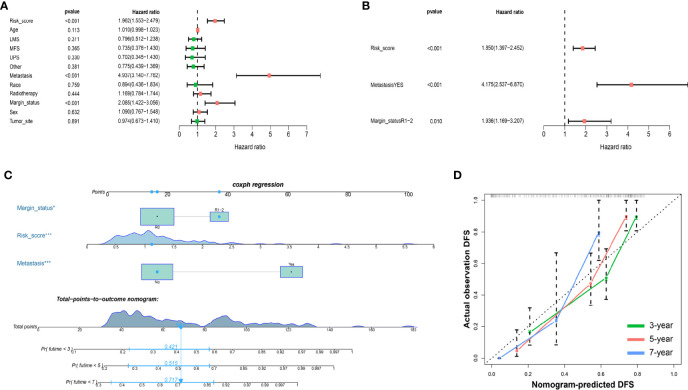
Nomograms based on DFS-related IMRGs for osteosarcoma patients. **(A)** Univariate Cox analysis of DFS-related variables. **(B)** Multivariate Cox analysis of DFS-related variables. **(C)** Establish a nomogram to predict the DFS of patients. **(D)** The calibration curve shows that using a nomogram to predict DFS is highly consistent with actual DFS. DFS, disease-free survival; IMRGs, iron metabolism-related genes.

To further determine the clinical value of the prognostic signature for DFS, Cox regression analysis was performed (**Figures 7A, B**). In the univariate Cox analysis, the results showed that metastasis, margin status, and risk score were significantly associated with the DFS of sarcoma patients. Multivariate Cox regression analysis revealed that metastasis, margin status, and risk score can independently predict the DFS of patients with sarcomas. Based on these independent prognostic factors, a nomogram for predicting DFS in sarcoma patients was constructed (**Figure 7C**), and the C-index reached 0.763 (95% CI: 0.706–0.820). The calibration plots indicated great predictive performance (**Figure 7D**).

## Discussion

Currently, it is widely recognized that the traditional staging system cannot adequately predict the prognosis of cancer patients (14–17). Biomarkers related to tumor diagnosis and prognosis urgently need to be developed. Previous studies have found that iron is highly required at all stages of tumor development (18). Iron metabolism pathways, including processes of uptake-export, storage, and regulation, may be abnormally regulated during cancer progression (19). For example, transferrin receptor 1 (TFR1) is involved in the regulation of iron uptake and cell growth, is abnormally expressed in tumors and is closely related to tumor proliferation and metastasis (20–22). However, current studies mainly focus on the role of iron metabolism in cancer development and treatment and rarely discuss the role of iron metabolism genes in cancer prognosis (23, 24).

In the present study, based the expression pattern of IMRGs, three clusters of sarcoma subgroups were identified by consensus clustering analysis. The results revealed significant differences in DFS, TMB and tumor microenvironment between the three clusters. Cluster 1 patients with lower TMB had a better prognosis than those in the other two clusters. Previous studies have shown that iron plays a critical role in the reprogramming of the TME (18, 25). The TME is abundant with a variety of leukocytes, of which macrophages dominate. The density of M2 phenotype macrophages is correlated with poor prognosis (26). However, M1 phenotype macrophages generally have antitumor properties (27). Our results showed that patients in cluster 1 had the lowest infiltration level of M2 macrophages, while the level of M2 macrophage infiltration in cluster 2 was the same as that in cluster 3, but cluster 2 had the highest infiltration level of M1 macrophages. We speculate that this may be the reason why the prognosis of patients in cluster 2 is better than that in cluster 3. This result seems to be consistent with previous conclusions. M1 macrophages show iron-accumulating properties, while M2 macrophages show iron-releasing properties (28). The possible reason for this difference is that the iron released by M2 macrophages can aggravate abnormal iron metabolism in tumor cells. The mechanism may be related to increased iron export through FPN and increased iron-related proteins (18). Undoubtedly, targeting iron metabolism in M2 macrophages is a promising therapeutic strategy to suppress tumor growth.

Disordered iron metabolism is one of the hallmarks of tumors, and iron metabolism is significantly associated with the prognosis of cancer patients. Therefore, the construction of a novel signature using IMRGs is of great significance to provide new therapeutic targets and improve prognosis in patients with sarcomas. In our work, we performed univariate Cox regression to identify IMRGs related to the clinical outcome of patients with sarcomas. Nine IMRGs were found to be significantly related to the clinical outcome of sarcomas. Finally, LASSO regression analysis and multivariate Cox regression analysis were conducted, and five IMRGs (ABCB7, NCAO4, SFXN1, SLC25A28, SLC48A1) were included in the risk scoring model for predicting OS. ABCB7 is a mitochondrial iron transporter, and the expression of ABCB7 is associated with the prognosis of glioma patients. The loss of ABCB7 not only reduces the invasiveness of tumor cells but also results in cell death through dysregulated intracellular iron circulation and mitochondrial ROS generation (29). NCAO4 is a selective cargo receptor that mediates the autophagic degradation of ferritin (30). In prostate cancer, NCOA4*α* acts as a tumor suppressor, while NCOA4*β* expression is correlated with proliferation and invasion (31). The SFXN1 gene is associated with mitochondrial function and iron transport. The latest findings indicate that SFXN1 is a mitochondrial serine transporter required for one-carbon metabolism. Because a crowd of malignancies depends on the one-carbon units produced from serine for rapid proliferation and SFXN1 is expressed in many cancers, SFXN1 may play a special role in the proliferation of cancer (32). The SLC48A1 gene encodes an iron transporter that appears to transport heme from the endosome into the cytosol. *In vivo* and *in vitro* experiments have shown that overexpression of the SLC48A1 gene contributes to increased iron uptake, resulting in increased oxygen consumption and ATP production, which ultimately promotes the proliferation of NSCLC (33). The SLC25A28 gene encodes a mitochondrial iron uptake transporter (Mfrn2), which participates in As2O3-induced cell killing in glioma (34). Combined with these studies, we assumed that the iron metabolism-related signature for OS can accurately predict the clinical outcome of sarcoma patients. Subsequent research further confirmed that the multigene signature for OS is an independent prognostic factor for patients with sarcomas. Risk stratification by risk score showed that patients in the high-risk subgroup had a shorter OS than those in the low-risk subgroup. The good predictive performance of the multigene signature for OS we constructed was shown through the validation of the training set and validation set.

In addition, we also established a multigene signature for DFS using IMRGs (NCOA4, ISCU, SLC25A37, SLC48A1) and validated it through a training set and validation set. The multigene signature for DFS can independently and precisely predict the prognosis of sarcoma patients. The role of ISCU is to catalyze the assembly of iron-sulfur clusters, which are essential for the function of aconitase (a member of the tricarboxylic acid cycle) and mitochondrial ETC complexes I, II and III. In addition, the high expression of ISCU is related to the good prognosis of many kinds of tumors (35). The degradation of SLC25A37 can be mediated by the PINK1-PARK2 pathway to increase the accumulation of iron in mitochondria, which leads to the activation of the inflammasome in tumor cells. In patients with pancreatic cancer, the high expression of SLC25A37 is associated with poor prognosis (36, 37). Interestingly, NCOA4 and SLC48A1 are also included in the multigene signature for DFS, which implies that these two genes may play a more important role in the progression of sarcomas. Based on existing reports, the mechanism of these two genes in sarcoma is still unclear and requires follow-up research for further exploration. Our research is the first to use a large database to establish two signatures related to iron metabolism for predicting the prognosis of sarcoma patients, which undoubtedly provides a new treatment strategy for the treatment of sarcoma patients.

Last, our research also has some limitations. First, this is a retrospective study, so there may be biases in the selection of variables, resulting in a loss of data accuracy. Second, the prediction model constructed in this study is based on the estimation of the survival function after comprehensive analysis of various influencing factors on the premise of big data analysis. However, the prediction of OS and DFS of patients is restricted by the current medical level, so it is suggested that the prediction model constructed in this study should be included in future clinical trials, and further prospective verification has been carried out. Finally, further experimental verification is needed in the future to reveal the potential mechanism of IMRGs in sarcoma.

## Conclusion

In summary, our research systematically demonstrated that IMRGs were significantly associated with the TME. Then, we constructed two multigene prognostic signatures for OS and DFS that can both accurately predict the prognosis of sarcoma patients and provide new treatment strategies for sarcomas.

## Data Availability Statement

Publicly available datasets were analyzed in this study. This data can be found here: https://portal.gdc.cancer.gov/) and https://www.ncbi.nlm.nih.gov/geo/.

## Author Contributions

YX conceived and designed the study. YD, CS, TX, and YS performed the analysis. JL and CH wrote the paper. XT, HH, ZZ, JG, and YX reviewed and edited the manuscript. All authors read and approved the manuscript. All authors contributed to the article and approved the submitted version.

## Funding

This study was supported by the Taishan Scholar Project of Shandong Province, China [No. ts20190985].

## Conflict of Interest

The authors declare that the research was conducted in the absence of any commercial or financial relationships that could be construed as a potential conflict of interest.

## Supplementary Material

Supplementary Figure 1 LASSO analysis of IMRGs related to OS and DFS. **(A, C)** Draw a dashed vertical line with the best value by using the minimum standard. **(B, D)** LASSO coefficient curves of IMRGs related to candidate OS **(B)** and DFS **(D)**. LASSO, the smallest absolute contraction and selection operator; IMRGs, iron metabolism-related genes; OS, overall survival; DFS, disease-free survival.
