# Supplementary figures and images for: Identification of Iron Metabolism-Related Gene Signatures for Predicting the Prognosis of Patients With Sarcomas
Source: Front Oncol. 2021 Jan 7;10:599816. doi: 10.3389/fonc.2020.599816 (PMC7817539; doi:10.3389/fonc.2020.599816)

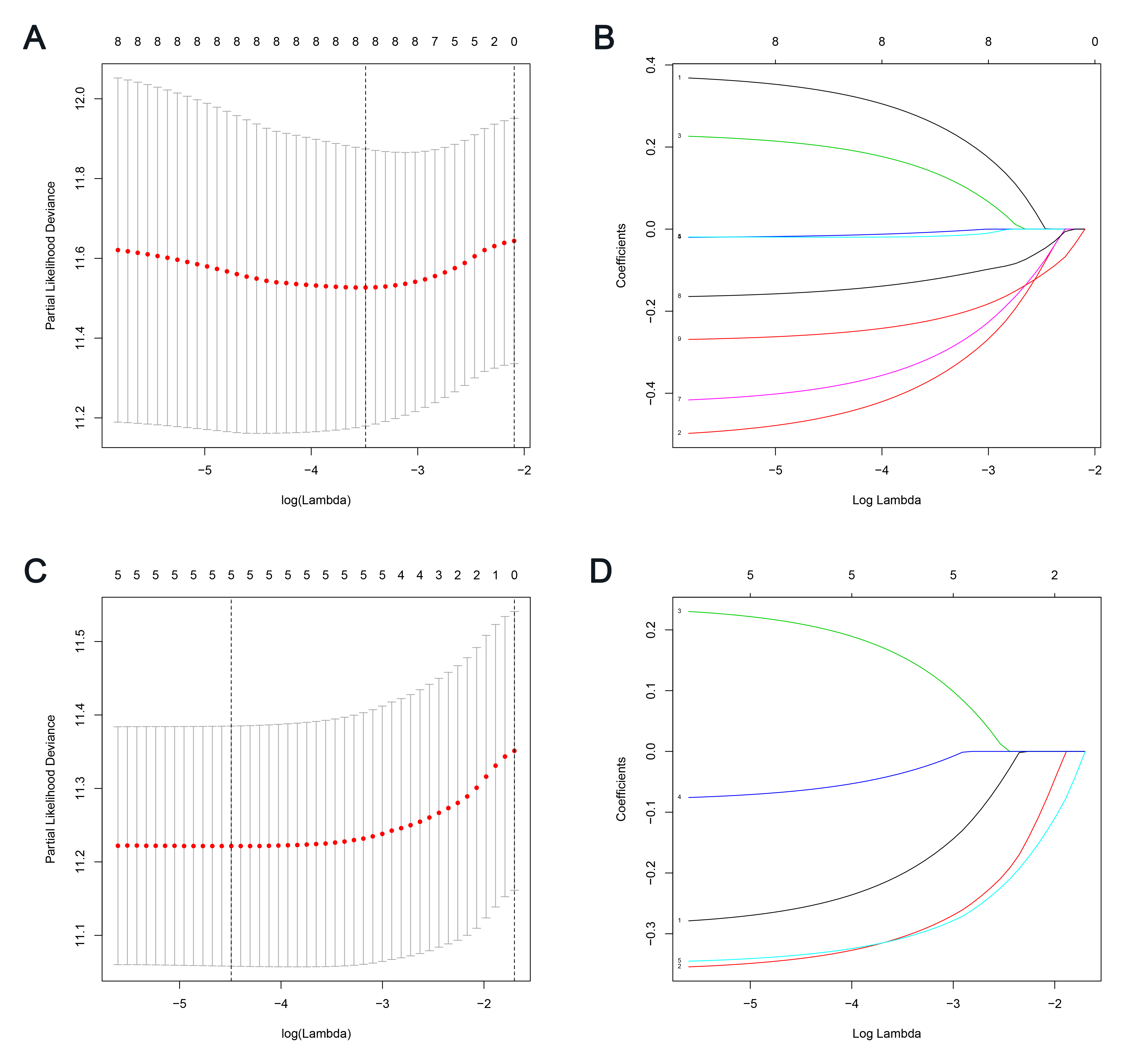

Supplement: Supplementary Figure 1 — LASSO analysis of IMRGs related to OS and DFS. (A, C) Draw a dashed vertical line with the best value by using the minimum standard. (B, D) LASSO coefficient curves of IMRGs related to candidate OS (B) and DFS (D). LASSO, the smallest absolute contraction and selection operator; IMRGs, iron metabolism-related genes; OS, overall survival; DFS, disease-free survival. [file Image_1.tif]
